# Eosinophilic pneumonia associated with bleomycin in a patient with mediastinal seminoma: a case report

**DOI:** 10.1186/1752-1947-4-126

**Published:** 2010-04-29

**Authors:** Sanjaykumar Hapani, David Chu, Shenhong Wu

**Affiliations:** 1 Division of Medical Oncology, Department of Medicine, Stony Brook University, Stony Brook, New York, USA

## Abstract

**Introduction:**

Lung toxicities resulting from the chemotherapeutic agent bleomycin encompass a variety of pathological changes, including bronchiolitis obliterans organizing pneumonia, interstitial pneumonitis and progressive interstitial fibrosis. We report a rare case of eosinophilic pneumonia associated with bleomycin.

**Case presentation:**

A 44-year-old Hispanic man with a primary mediastinal seminoma complicated by superior vena cava syndrome underwent treatment with four cycles of bleomycin, etoposide and cisplatin. He had a complete positive response to the chemotherapy. However, three months after treatment he presented with shortness of breath and severe hypoxemia associated with peripheral eosinophilia. Computed tomography showed bilateral diffuse interstitial infiltrates that were refractory to antibiotic treatment. A lung biopsy showed eosinophilic pneumonia. He was subsequently treated with high-dose prednisone, resulting in a complete resolution of his symptoms and lung infiltrates.

**Conclusion:**

This case illustrates that eosinophilic pneumonia may be a late sequela of bleomycin toxicity, and may respond dramatically to steroid treatment.

## Introduction

Bleomycin is an antineoplastic agent derived from *Streptomyces verticillus*, and is widely used in the treatment of testicular carcinoma, Hodgkin's and non-Hodgkin's lymphoma as well as squamous cell carcinomas of the head and neck. However it has well-known pulmonary toxicities, including diffuse alveolar damage, bronchiolitis obliterans organizing pneumonia (BOOP), interstitial pneumonitis, and progressive interstitial fibrosis [1]. This report illustrates a rare case of severe bleomycin-associated eosinophilic pneumonia (EP) that responded to steroid treatment.

## Case presentation

A 44-year-old Hispanic man was diagnosed in October 2006 with a primary mediastinal seminoma complicated by superior vena cava (SVC) syndrome. He was started on a first-line systemic therapy of bleomycin, etoposide and cisplatin (BEP). Bleomycin (30 units) was administered on days 2, 9 and 16; etoposide (100 mg/m^2 ^intravenously) on days 1 to 5; and cisplatin (20 mg/m^2 ^intravenously) on days 1 to 5 every three weeks for a total of four cycles. The total cumulative bleomycin dosage was 360 units with the last dose of bleomycin administered on 29 December 2006. Following chemotherapy, the patient achieved a complete response to treatment with resolution of the SVC syndrome. His anterior mediastinal mass decreased substantially in size, with a complete normalization of the standardized uptake value (SUV) by computed tomography (CT) and positron emission tomography (PET); his beta human chorionic gonadotropin (β-HCG) level decreased from 5452 to an undetectable level; and his alpha fetoprotein (AFP) level remained within the normal range. He tolerated the chemotherapy without any adverse side effects.

**Figure 1:**
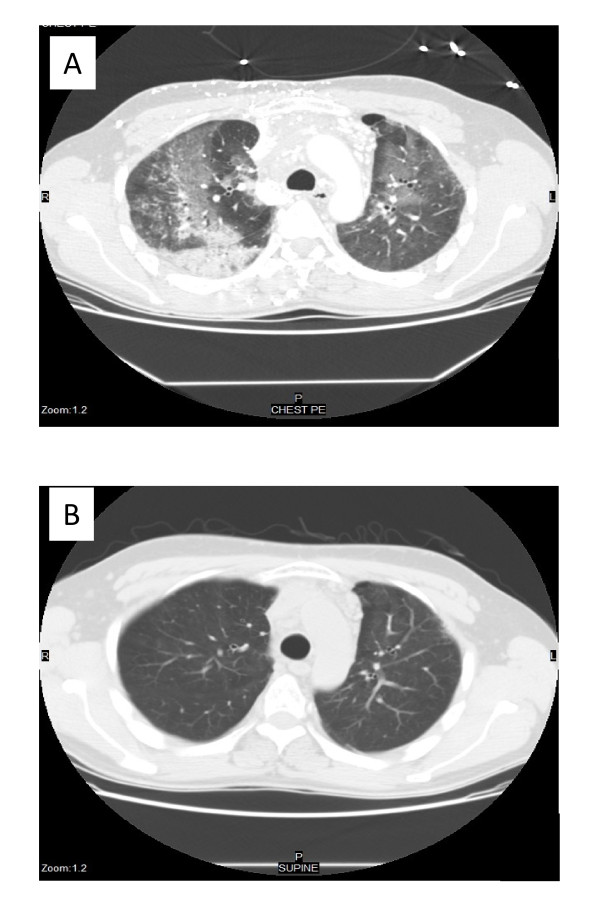
**Pulmonary infiltrates before and after steroid treatment**. (A) Computed tomography (CT) of chest with intravenous contrast in March 2007 showing right upper lobe, right middle lobe and left lung with patchy ground-glass opacities. (B) CT of chest one month after steroid treatment showing complete resolution of ground-glass opacities in both lung fields.

**Figure 2:**
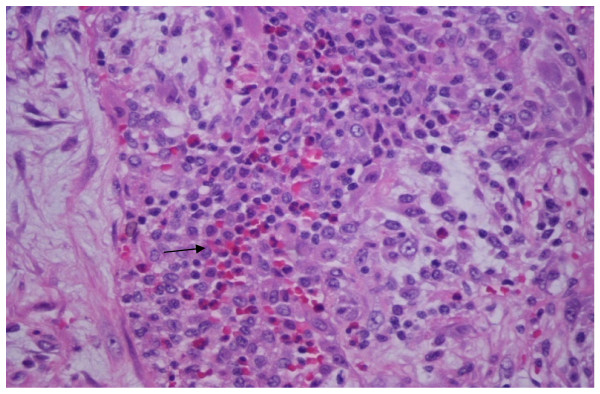
**Histology of pulmonary lesions**. Hematoxylin and eosin stain was used for the lung biopsy, original magnification, 400×. Arrow points to eosinophils with pink color; alveoli are infiltrated with inflammatory cells, mainly eosinophils, lymphocytes and neutrophils. There is no evidence of vasculitis or alveolar hemorrhage. There is some fibrous tissue in the periphery. Note that only distal airways are involved with sparing of proximal airways.

Three months after the treatment, he presented at the emergency department at Stony Brook University Medical Center, having suffered from progressive shortness of breath for three days but without any other obvious precipitating factors. He was not on any medication and he did not have any gastrointestinal symptoms. Physical examination revealed tachycardia, tachypnea, hypoxia and decreased breath sounds with fine crackles bilaterally. Chest X-ray showed a right lower lobe infiltrate. Interestingly, his eosinophil count had increased from a baseline level of 2% to 10%, although his total white blood cell count was within the normal range. Subsequent CT of his chest showed extensive patchy ground-glass opacities in the right upper lobe, middle lobe and left lung without evidence of any pulmonary embolism (Figure 1A). He was treated with ceftriaxone and azithromycin empirically for community acquired pneumonia. Because he did not respond to a four-day course of the antibiotic treatment and showed worsening dyspnea, our patient was admitted to the medical intensive care unit, and underwent a thoracoscopic right middle lobe wedge biopsy to investigate possible bleomycin-induced lung toxicity. Pathological examination of the lung tissue revealed severe widespread organizing pneumonia with accompanying eosinophil-rich inflammatory infiltrates (Figure 2). Cultures and stains of the tissue showed negative for any infectious agents including *Mycobacterium tuberculosis*, viral, fungal or *Pneumocystis jirovecii *infection. There was also no evidence of seminoma recurrence.

Our patient was started on oral prednisone 70 mg daily, and his respiratory status slowly improved over the next few days. Treatment continued with a high-dose prednisone for one month. A follow-up CT of his chest revealed a complete resolution of the bilateral ground-glass opacities (Figure 1B). Furthermore, his peripheral eosinophilia level decreased from 10% to <2%. The prednisone was then tapered off over a period of three months. Our patient has remained symptom-free and disease-free for two years.

## Discussion

The association between bleomycin and EP has not been well established. Only three cases of bleomycin-induced EP with a self-limiting clinical course have been described in the literature so far [2]. Our patient demonstrates a severe case of EP associated with the use of bleomycin that responded well to steroid treatment.

Bleomycin-induced lung injury occurs in 3-5% of patients treated with the drug, but fatal effects are rare [3,4]. Patients who suffer from lung and skin toxicities may be deficient in a bleomycin hydrolase enzyme [5]. Risk factors for bleomycin toxicity include older age, cigarette smoking, prior radiotherapy, oxygen therapy, a cumulative dose of bleomycin greater than 450U, an intravenous route of administration of the drug, and an underlying or worsening renal insufficiency [6-10]. There are several known pulmonary-related adverse effects of bleomycin including diffuse alveolar damage, pneumonitis, BOOP and pulmonary fibrosis. However, none of these changes are pathognomonic of bleomycin toxicity.

The relationship between EP and bleomycin has been suggested by others in earlier case studies. Yousem *et al*. described three cases of bleomycin-associated EP with few symptoms and a self-limiting clinical course, for which corticosteroids were not used to treat the condition [2]. Holoye *et al*. described three cases of bleomycin-associated hypersensitivity pneumonitis with patchy eosinophilic infiltrates that fitted the spectrum of EP [11].

Eosinophilic pneumonia is an inflammatory, reactive pulmonary process with multiple etiologies including systemic diseases such as Churg-Strauss disease, and parasitic diseases. It can also be caused by a variety of drugs including antibiotics, non-steroidal anti-inflammatory drugs (NSAIDs), anti-convulsants, anti-depressants, angiotensin converting enzyme inhibitors, inhaled toxins such as cigarette smoke, and chemotherapy [12]. Drug-induced EP may be considered as a possible hypersensitivity reaction. Afflicted patients may complain of a wide range of symptoms ranging from flu-like symptoms to shortness of breath, cough, weight loss and fever. The disease can manifest itself as early as days to some weeks after exposure to the inciting etiology, or as a late sequela a few months later. Our patient developed respiratory symptoms three months after completing the bleomycin treatment, suggesting a late sequela.

A definitive diagnosis of EP is made when there is evidence of a parenchymal patchy infiltrate on a chest X-ray, peripheral blood eosinophilia, and increased eosinophils in either lung biopsy tissue or bronchoalveolar lavage (BAL) fluid (usually >25%) [12]. A BAL or lung biopsy can be undertaken, depending on the location of the ground-glass opacity and its accessibility. Histological examination of the lung tissue should reveal an accumulation of eosinophils and macrophages in the alveoli; the alveolar septa are thickened and infiltrated by eosinophils, lymphocytes and macrophages [13]. CT may be helpful as a 'road map' for a potential biopsy or to give a better view of the location of the opacities, but it is not necessary to confirm a diagnosis.

The toxicity related to bleomycin is two-fold. The primary event is thought to be an injury to the endothelial cells leading to fluid leakage and pulmonary hemorrhage. Subsequently it causes direct toxicity to type 1 pneumocytes which can lead to death, and secondary hyperplasia of type 2 pneumocytes [9].

There is no proven effective treatment for bleomycin-induced pneumonitis, although corticosteroids are widely used [5]. For patients with asymptomatic EP, it could be a self-limiting condition, and close observation may be a sufficient treatment, as shown by Yousem *et al*. [2]. However, steroids remain the core treatment of EP. In our case of severe bleomycin-associated EP, high-dose prednisone therapy led to the rapid resolution of the respiratory symptoms as well as of the radiographic abnormalities. It is thought that corticosteroids induce healing by apoptosis of the eosinophils [12]. Our case therefore suggests that there is a role for steroids in the treatment of bleomycin-associated EP.

## Conclusion

We have presented a case of severe EP associated with the use of bleomycin. It manifested as a late sequela of the bleomycin treatment and responded to high-dose steroid treatment. Eosinophilic pneumonia must be considered in the differential diagnosis of pulmonary infiltrates after bleomycin therapy; and it can respond well to steroids in severe cases.

## Abbreviations

AFP: alpha fetoprotein; β-HCG: beta human chorionic gonadotropin; BOOP: bronchiolitis obliterans organizing pneumonia; BEP: bleomycin, etoposide, cisplatin; CT: computed tomography; EP: eosinophilic pneumonia; NSAID: non-steroidal anti-inflammatory drug; PET: positron emission tomography; SVC: superior vena cava; SUV: standardized uptake value.

## Consent

Written informed consent was obtained from the patient for publication of this case report and any accompanying images. A copy of the written consent is available for review by the Editor-in-Chief of this journal.

## Competing interests

The authors declare that they have no competing interests.

## Authors' contributions

SW conceived the idea of the study. SH collected the data. All the authors contributed to the writing, and read and approved the final manuscript.
